# Increased serum C1q-binding adiponectin complex to total-adiponectin ratio in men with multi-vessel coronary disease

**DOI:** 10.1186/1758-5996-6-64

**Published:** 2014-05-27

**Authors:** Ken Kishida, Yasuhiko Nakagawa, Hironori Kobayashi, Koji Yanagi, Tohru Funahashi, Iichiro Shimomura

**Affiliations:** 1 Department of Metabolic Medicine, Graduate School of Medicine, Osaka University, Suita, Osaka, Japan; 2 Kishida Clinic, Toyonaka, Osaka, Japan; 3 Department of Research and Development, Diagnostic Division, Otsuka Pharmaceutical Co., Ltd, Tokushima, Japan; 4 Department of Cardiology, Kenporen Osaka Central Hospital, Osaka, Japan; 5 Department of Metabolism and Atherosclerosis, Graduate School of Medicine, Osaka University, Suita, Osaka, Japan

**Keywords:** Adiponectin, C1q, C1q-binding adiponectin, Coronary artery disease, Angiographic coronary vessel

## Abstract

**Background:**

Adiponectin plays a role as a positive contributor to the stabilization of atherosclerotic plaques. Circulating total adiponectin (Total-APN) levels associates with the number of coronary vessels in men with coronary artery disease (CAD). We recently reported that adiponectin binds to C1q in human blood, and serum C1q-binding adiponectin (C1q-APN) /Total-APN levels are associated with CAD in type 2 diabetic subjects. The present study investigated the relationship between circulating C1q-APN levels and the number of angiographic coronary artery vessel in male subjects.

**Methods:**

The study subjects were 53 male Japanese patients who underwent diagnostic coronary angiography. Blood total adiponectin (Total-APN), high-molecular weight adiponectin (HMW-APN), C1q-APN and C1q were measured by enzyme-linked immunosorbent assays.

**Results:**

Serum C1q-APN/Total-APN ratio significantly increased in subjects with single and multi-vessel coronary diseases (p = 0.029 for trend, the Kruskal-Wallis test). However, serum Total-APN, HMW-APN, C1q-APN and C1q levels did not correlate with number of diseased coronary vessels.

**Conclusion:**

Serum C1q-APN/Total-APN ratio progressively increases in men with single and multi-vessel coronary disease.

**Trial registration:**

UMIN000002997

## Background

Vulnerable atheromatous plaques lead to coronary plaque disruption with superimposed thrombosis [1], which is often manifested as angiographically complex lesions [2]. Adiponectin, an adipocyte-derived blood protein [3] is present abundantly in injured arteries [4,5] and also suppresses macrophage foam cell transformation [6,7] and increases the expression of tissue inhibitor of metalloproteinase-1 in monocyte-derived macrophages through induction of interleukin-10 [8], suggesting that adiponectin may be a positive contributor to the stabilization of atherosclerotic plaques. Previous studies reported that low circulating total-adiponectin (Total-APN) levels were associated with coronary lesion complexity and the numbers of coronary vessels in subjects with coronary artery disease (CAD) [9-12]. Yu et al. reported that Total-APN levels decreased progressively as the number of diseased coronary arteries increased, although the differences in Total-APN levels among each coronary lesion severity were not statistically significant [10].

We reported recently that adiponectin bound with C1q in human blood, and also developed a system to measure human serum C1q-binding adiponectin (C1q-APN) [13]. Serum C1q-APN/Total-APN ratio correlated with the metabolic syndrome in male subjects [13], and with polyvascular diseases and stable CAD in type 2 diabetics [14,15]. However, the relationship between serum C1q-APN levels and the number of diseased coronary arteries remains to be elucidated. The hypothesis tested in the present study is that C1q-APN may be associated with the presence of multi-vessel coronary disease. The main investigation and results in this study were not in the coronary stenosis morphology but only in the number of diseased vessels.

## Methods

### Participants

The study (#UMIN 000002997) subjects were consecutive 53 male Japanese admitted-patients who underwent diagnostic coronary angiography for suspected CAD following chest pain and/or ischemic changes on the electrocardiogram, and also underwent measurement of fat distribution by computed tomography scan for measurement of adipose tissues that was classified to visceral and subcutaneous fat tissue compartment at Kenporen Osaka Central Hospital and Department of Metabolic Medicine, Osaka University Hospital between April and September 2009. Patients treated with pioglitazone, which is known to increase serum levels of Total-APN [16] and C1q-APN [17], and those with renal dysfunction (creatinine >1.5 mg/dL) [18] were excluded from the study. The Medical Ethics Committees of Osaka University and Kenporen Osaka Central Hospital approved the protocol of the study. All participants were Japanese and each gave a written informed consent.

### Angiographic morphology of coronary stenosis

Coronary stenosis was assessed morphologically according to the American College of Cardiology/American Heart Association (ACC/AHA) [19], and patients were classified into three groups, as follows: Non-CAD group (control), patients with no stenosis; Single-vessel group, patients with > 75% stenosis of one vessel; Multi-vessels group, patients with > 75% stenosis of two or more vessels. Angiographic evaluations were performed independently by 2 cardiologists who were blinded to the clinical features of the patients and, in case of disagreement, the decision was based on the judgment of a third, more experienced observer. The interobserver reproducibility for morphologic assessment of coronary lesions was 90%.

### Anthropometry and laboratory tests

Anthropometric variables [height and weight] were measured in the standing position and body mass index (BMI) was calculated [=weight (kg)/height (m)^2^]. Visceral fat area (VFA) and subcutaneous fat area (SFA) were measured manually on computed tomography scan at the umbilical level according to our laboratory methods [20]. Systolic and diastolic blood pressures were measured with a standard mercury sphygmomanometer on the left or right arm in the supine position after at least 10-minute rest.

Venous blood samples were collected in the morning after overnight fast for measurement of serum creatinine, lipids, glucose, and HbA1c (Japan Diabetes Society [JDS]). The value of HbA1c (%) was estimated as the National Glycohemoglobin Standardization Program (NGSP) equivalent value (%), calculated by the formula HbA1c (%) = HbA1c (JDS,%) + 0.4%. For the purpose of the present study, serum samples that were obtained at baseline from each participant were stored promptly at –20°C. After thawing the samples, serum levels of Total-APN and high-molecular weight-adiponectin (HMW-APN) were measured by enzyme-linked immunosorbent assay (ELISA) kits (Human adiponectin ELISA kit, Human HMW-adiponectin ELISA kit, Otsuka Pharmaceutical Co. Tokushima, Japan) [3,21]. Serum levels of C1q-APN (units (U) /mL) and C1q (μg/mL) were measured by our in-house ELISA, as reported previously by our group [13]. The intra- and inter-coefficients of variation (CV) for C1q-APN ELISA are below 4.6% and 6.7%, respectively. The intra- and inter-CV for C1q ELISA are below 4.6% and 5.0%, respectively.

Hypertension was defined as systolic blood pressure ≥140 mmHg, and/or diastolic blood pressure ≥90 mmHg, or current treatment for hypertension. Diabetes mellitus was defined according to the World Health Organization criteria [22], and/or current treatment for diabetes mellitus. Dyslipidemia was defined as low-density lipoprotein-cholesterol concentration of ≥140 mg/dL, triglyceride concentration ≥150 mg/dL, high-density lipoprotein-cholesterol concentration <40 mg/dL, and/or treatment for dyslipidemia.

### Statistical analysis

Data are presented as mean ± SEM. Differences in frequencies were examined by the χ^2^ test. Differences among groups were compared by one- or two-way analysis of variance (ANOVA) with Fisher's protected least significant difference test for multiple-group analysis. Differences in each adiponectin parameter in numbers of vessels were analyzed by the Kruskal-Wallis test. In all cases, *p* values <0.05 were considered statistically significant. All analyses were performed with the JMP Statistical Discovery Software 9.0 (SAS Institute, Cary, NC).

## Results

**Figure 1:**
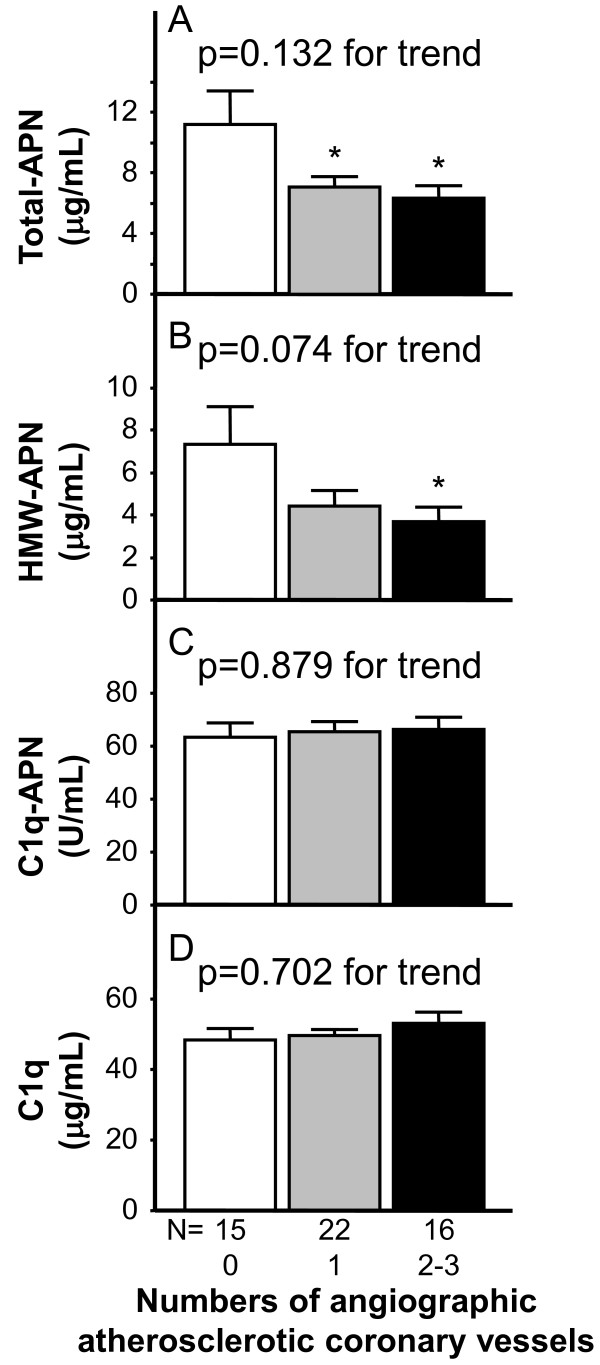
**Circulating levels of Total-APN (A), HMW-APN (B), C1q-APN (C), and C1q (D) in the study population according to the number of coronary vessels.** Differences in each adiponectin parameter and C1q in numbers of vessel were analyzed by the Kruskal-Wallis test. Differences among groups were compared by one- or two-way analysis of variance (ANOVA) with Fisher’s protected least significant difference test for multiple-group analysis. *p < 0.05, compared with the Non-CAD group (number of coronary vessel = 0).

Single and multiple vessel disease was identified in 72% (none/single/double/triple = 15/22/13/3). Table 1 summarizes the characteristics of the participating subjects, according to the number of diseased coronary vessels; Non-CAD group, Single-vessel group, Multiple-vessels group. There were no significant differences of age, BMI, VFA and creatinine among three groups. Serum Total-APN levels were significantly lower in single-vessel and multi-vessels groups than in Non-CAD group (Figure 1A). However, there was no significance of serum C1q-APN and C1q levels among three groups (Figure 1B-D).

**Table 1:** Baseline characteristics of the subjects enrolled in the present study

	**Non-CAD group**	**Single-vessel group**	**p value (Non-CAD group versus Single-vessel group)**	**Multi-vessels group**	**p value (Non-CAD group versus Multi-vessels group)**	**p value (Single-vessel group versus Multi-vessels group)**
Number	15	22		16		
Age, years	65 ± 2	65 ± 2	0.910	66 ± 2	0.889	0.817
Body mass index, kg/m^2^	25.0 ± 0.9	23.8 ± 0.7	0.666	25.6 ± 1.0	0.359	0.135
Visceral fat area, cm^2^	111 ± 14	121 ± 14	0.205	123 ± 14	0.255	0.898
Subcutaneous fat area, cm^2^	137 ± 18	138 ± 12	0.341	141 ± 11	0.394	0.918
Smoking (none-/ex-/current-smoker), n	8/5/2	9/5/8	0.566	3/4/9	0.149	0.230
Diabetes mellitus, n	9	11	0.644	9	0.879	0.793
Sulfonyl ureas/glinides/biguanides/alpha glucosidase inhibitors/Insulin, n	5/0/1/1/2	7/5/6/1/2		4/0/1/4/2		
Hypertension, n	13	16	0.436	11	0.392	0.886
Calcium channel antagonists/angiotensin receptor blockers/β-blockers/diuretics, n	8/2/2/0	13/12/4/2		6/8/6/2		
Dyslipidemia, n	7	14	0.430	10	0.525	0.965
Statins/fibrates/ezetimibe/cholestimide, n	5/1/0/0	10/0/1/1		9/1/0/0		
Anti-platelet drugs (aspirin/ticlopidine/clopidogrel), n	4/0/2	22/0/22		16/6/10		
Family history of CAD, n	2	2	0.753	5	0.392	0.116
Systolic blood pressure, mmHg	134 ± 4	137 ± 4	0.808	139 ± 5	0.981	0.802
Diastolic blood pressure, mmHg	78 ± 2	74 ± 2	0.135	77 ± 3	0.586	0.407
Hemoglobin A1c (NGSP),%	6.9 ± 0.5	6.4 ± 0.2	0.255	6.6 ± 0.4	0.661	0.519
LDL-C, mg/dL	123 ± 6	110 ± 7	0.193	113 ± 9	0.789	0.132
Triglyceride, mg/dL	124 ± 16	152 ± 19	0.290	125 ± 12	0.969	0.273
HDL-C, mg/dL	61 ± 4	57 ± 4	0.352	48 ± 3	0.005*	0.139
Creatinine, mg/dL	0.82 ± 0.04	0.87 ± 0.06	0.880	0.82 ± 0.02	0.475	0.510
Target lesions (LMCA/LAD/LCX/RCA), n	-	0/20/1/1		1/40/13/2		
ACC/AHA (Type A/B/C)	-	6/12/4		5/7/4		
Procedures (PCI/CABG), n	-	22/0		15/1		

Data are mean ± SEM, or number of subjects analyzed. Differences among groups were compared by one- or two-way analysis of variance (ANOVA) with Fisher's protected least significant difference test for multiple-group analysis. Differences in frequencies were examined by the χ^2^ test. CAD, coronary artery disease; HDL-C, high-density lipoprotein-cholesterol; LDL-C, low-density lipoprotein-cholesterol; LMCA, left main coronary artery; LAD, left anterior descending artery; LCX, left circumflex artery; RCA, right coronary artery; PCI, percutaneous coronary intervention; CABG, coronary artery bypass graft.

**Figure 2:**
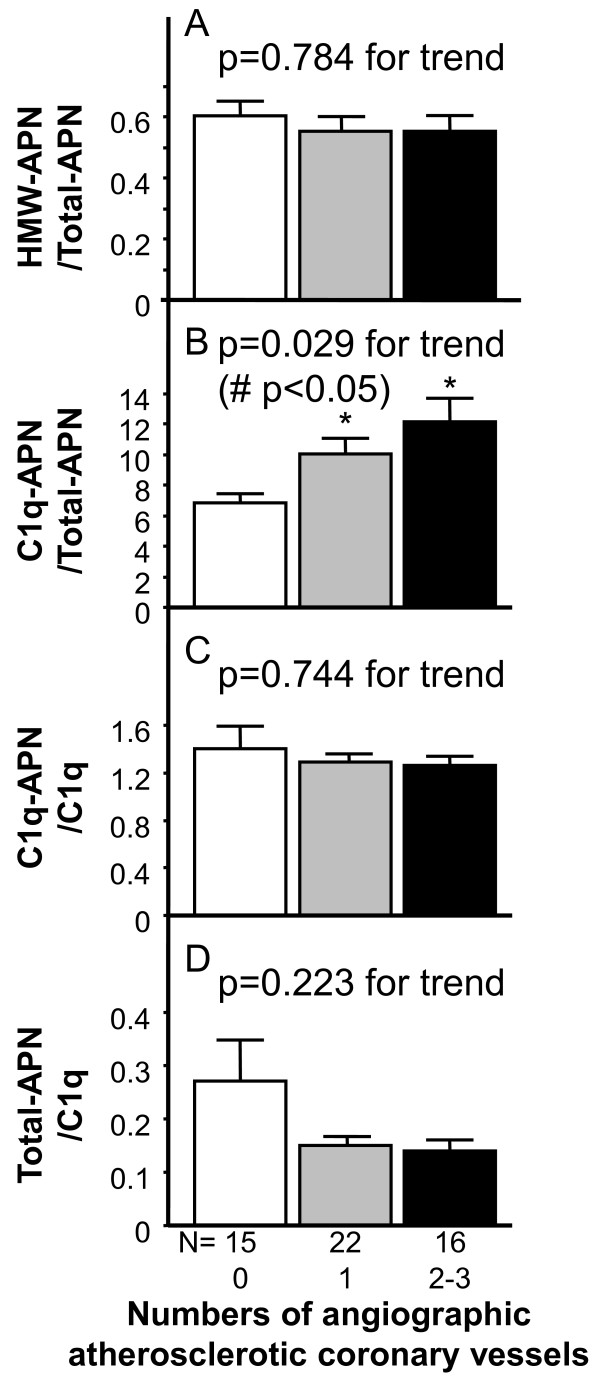
**Circulating levels of HMW-APN/Total-APN (A), C1q-APN/Total-APN (B), C1q-APN/C1q (C), and Total-APN/C1q (D) in the study population according to the number of coronary vessels.** Differences in each adiponectin parameter in numbers of vessel were analyzed by the Kruskal-Wallis test (# p < 0.05). Differences among groups were compared by one- or two-way analysis of variance (ANOVA) with Fisher's protected least significant difference test for multiple-group analysis. *p < 0.05, compared with the Non-CAD group (number of coronary vessel = 0).

Our groups reported that serum C1q-APN/Total-APN ratio correlated with polyvascular diseases and stable CAD in type 2 diabetics [14,15]. It is important to consider not only the absolute amount of adiponectin but also the levels of relative adiponectin forms in blood. Serum C1q-APN/Total-APN levels were significantly higher in single-vessel and multi-vessels groups than in Non-CAD group (*p < 0.05, Figure 2B). Serum C1q-APN/Total-APN ratio increased significantly in men with single and multi-vessel coronary diseases (#p < 0.05, the Kruskal-Wallis test, Figure 2B). However, serum HMW-APN/Total-APN, C1q-APN/C1q and Total-APN/C1q levels did not correlate with the number of coronary vessels (Figure 2A, C and D).

## Discussion

The present study represents the first report demonstrating that serum C1q-APN/Total-APN ratio increased progressively in men with single and multi-vessel coronary disease.

The present study demonstrated that serum Total-APN levels were significantly lower in single-vessel and multi-vessels groups than in Non-CAD group (Figure 1A), as reported previously [10,12], but did not decrease significantly with the increased numbers of atherosclerotic coronary vessels (p = 0.132 for trend, Figure 1A). Serum HMW-APN levels were also significantly lower in multi-vessels groups than in Non-CAD group (Figure 1B), and trended to decrease with the increased numbers of atherosclerotic coronary vessels (p = 0.074 for trend, Figure 1B). There was a strong positive relation of between serum Total-APN and HMW-APN levels in the present study (p < 0.0001, r = 0.93, data not shown), as reported previously [21,23]. There are no different characteristics of between Total-APN and HMW-APN. Interestingly, serum C1q-APN/Total-APN levels were not only significantly higher in single-vessel and multi-vessels groups than in Non-CAD group, but also they increased significantly with increased numbers of coronary vessels (Figure 2B). We speculate that adiponectin may be trapped by C1q, as adiponectin is trapped by cystatin C in renal failure [24], thereby leading to an inactive adiponectin form, C1q-APN. However, to date, the precise values for serum C1q-APN cannot be measured, because the proportion of blood adiponectin that forms protein complex with C1q remains unclear. If feasible, we may clarify whether C1q-APN is an inactive form of adiponectin.

Angiographic assessment of the morphology of coronary stenosis is well established and considered to be clinically useful for risk stratification of CAD patients [25,26]. Our results may indicate that high serum C1q-APN/Total-APN could perhaps be used to predict the occurrence of future cardiovascular events; however, further prospective studies are required to clarify this issue because we did not assess clinical outcomes in the present study. The present study did not investigate angiographic severity scores such as Jeopardy score, Gensini score or SYNTAX score, and coronary lesions using intravascular ultrasound. Therefore, further studies including vulnerability using intravascular ultrasound are required in the future.

## Conclusion

The present study indicates that serum C1q-APN/Total-APN ratio increased progressively in men with single and multi-vessel coronary disease. This study suggested serum C1q-APN/Total-APN might be a useful biomarker of coronary lesions in patients with CAD. Routine measurement of adiponectin in patients with lifestyle-related diseases is highly recommended [23]. Measurements of serum Total-APN plus C1q-APN may become instrumental to predict numbers of angiographic atherosclerotic coronary vessels.

### Study limitations

The present study has several limitations. First, all patients in this study were Japanese men and any differences from other ethnic groups are unknown. It is well-known that there is difference of circulating adiponectin levels in between males and females [27]. Further studies for females are required in the future. Second, the number of patients was relatively small. Based upon 80% power to detect statistically significant differences (p = 0.05; two-sided) as our group reported previously [13,14,28], a sample size of at least 15 patients in each group was required to demonstrate (total sample size = 45). Further a larger scale study is required in the future. Third, among patients with normal coronaries, coronary spastic patients with non-significant organic stenosis could not be excluded from the study, because they did not undergo intracoronary injection of acetylcholine chloride. There might be a patient selection bias. Fourth, this is a cross-sectional study, making it difficult to establish a cause-effect relationship. Further prospective studies should be conducted to analyze this relationship. Finally, the current study did not include the effects of data of each adiponectin parameter, such as pharmacological agents.

## Abbreviations

BMI: Body mass index; CAD: Coronary artery disease; C1q-APN: C1q-binding adiponectin; ELISA: Nzyme-linked immunosorbent assay; HMW-APN: High-molecular weight adiponectin; SFA: Subcutaneous fat area Total-APN, total-adiponectin; VFA: Visceral fat area.

## Competing interests

TF is a member of the “Department of Metabolism and Atherosclerosis”, a sponsored course endowed by Kowa Co. Ltd.. The company has a scientific officer who oversees the program. All other authors declare no competing interests. Human serum C1q-binding adiponectin complex assay is under patent application in Japan.

## Authors’ contributions

KK researched and analyzed the data, participated in the concept and design of the study, interpretation of data and reviewed/edited the manuscript. HK analyzed the data. YN and KY recruited the patients and collected the data. TF and IS contributed to the discussion. All authors read and approved the final version of the manuscript.
